# A Poroelastic Computational Modeling Framework Coupling Left Ventricular Cardiac Mechanics and Myocardial Perfusion

**DOI:** 10.1002/cnm.70219

**Published:** 2026-10-07

**Authors:** Haowei An, Vahid Ziaei‐Rad, Lik‐Chuan Lee

**Affiliations:** ^1^ Department of Mechanical Engineering Michigan State University East Lansing Michigan USA

**Keywords:** cardiac mechanics, coronary blood flow, coronary sinus interventions, myocardial perfusion, poroelasticity

## Abstract

We present a computational modeling framework coupling a poroelastic left ventricle (LV) with lumped‐parameter descriptions of the coronary and systemic circulations in a closed‐loop system. The finite element method, with P1‐P1‐P0 discretization of the displacement, Darcy flow and pore‐pressure fields, is used to solve the boundary value problem associated with the poroelastic formulation. We show that simulation of the isolated non‐contractile poroelastic LV can reproduce perfusion‐induced stiffening features found in measurements of pressure‐volume relationships with less than 1 mmHg error. Simulations coupling the poroelastic LV with systemic and coronary circulatory models also show that the framework can produce phasic changes in coronary flow rate and myocardial wall volume that agree with the experiments. Using the framework, we investigate the effects of coronary sinus (CS) resistance on myocardial perfusion. We show that increasing CS resistance attenuates coronary flow rate, increases myocardial pore pressure, and promotes myocardial swelling.

## Graphical abstract

A computational framework is developed to couple left ventricular poroelastic mechanics with coronary and systemic circulations. The model captures perfusion‐induced stiffening of the isolated heart, phasic coronary flow, and myocardial wall volume changes consistent with experiments. It further predicts that elevated coronary sinus resistance impairs perfusion, increases pore pressure, and promotes myocardial edema.

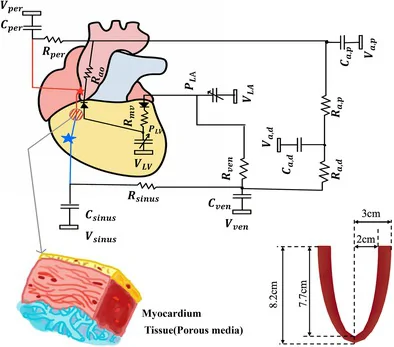

## 1 Introduction

Perfusion in the myocardium is tightly coupled to its mechanics, with both systems jointly controlling and affecting heart function. Mechanics and perfusion in the myocardial wall also interact bi‐directionally, where changes in one system affect the other and vice‐versa. As underscored in the seminal review by Spaan [1], coronary perfusion cannot be adequately described by flow or resistance alone but instead arises from the dynamic coupling between blood flow, vascular volume, and vascular compliance, all of which are profoundly influenced by myocardial contraction. Elucidating the interactions between myocardial wall mechanics and perfusion, and how they affect heart function is critical in developing a mechanistic understanding of different pathological conditions such as myocardial ischemia, as well as novel treatments such as coronary sinus occlusion (CSO) [2, 3].

Developed by Biot in the 1940s [4], the general theory of poroelasticity has been applied to describe the mechanical behavior of a wide range of materials, including (soft and hard) biological tissues, concrete and soil. In this theory, the material consists of a single or multiple fluid phases interspersed in “pores” within a solid skeleton. Fluid flow in the material is affected by deformation of the solid skeleton, and is usually described based on Darcy's law [5]. Because the myocardium is essentially a mixture of different constituents that include load bearing phases (e.g., collagen fibers and myocytes) and fluid phases (e.g., blood and interstitial fluid), the theory of poroelasticity provides an apt framework to describe the myocardial tissue mechanical behavior.

Computational models based on the poroelasticity theory have been developed to understand how myocardial perfusion affects wall stiffness and left ventricular (LV) function. Treating the myocardium as a (multi‐compartment) porous media, these models couple Darcy‐type perfusion with finite deformation cardiac mechanics to quantify how intramyocardial fluid pressure and volume fractions modulate effective tissue stiffness and ventricular performance [6, 7, 8, 9, 10]. The early models [6, 7] focus on the passive mechanical behavior of the myocardium and do not consider active myocardial contraction as well as the dynamic influence of contraction on coronary blood flow. Later work by Chapelle et al. [8] is mainly focused on theoretical development and does not consider the coupling of myocardial perfusion with coronary circulation. This limitation is addressed by the recent work of Barnafí Wittwer et al. [9] and Richardson et al. [10]. However, these studies do not systematically investigate the effects of mechanical parameters associated with distinct vascular compartments or the broader influence of upstream and downstream vascular properties on myocardial perfusion.

Building upon the strengths and limitations of these existing models, we seek here to develop a computational modeling framework based on the theory of poroelasticity to investigate the interaction between myocardial mechanics and perfusion. In this modeling framework, the LV myocardial wall is treated as a poroelastic medium that is coupled to a lumped‐parameter description of the coronary circulation. The LV cavity volume is also coupled to a lumped‐parameter description of the systemic circulation in a closed‐loop system. This setup enables us to consider the effects of upstream and downstream vascular parameters on myocardial perfusion over a full cardiac cycle. We show that the framework can produce features measured in the experiments and use the framework to investigate the effects of CSO on myocardial perfusion.

## 2 Methods

### 2.1 Formulation and Governing Equations of the Poroelastic Myocardium

The myocardium is modeled as a fully saturated poroelastic mixture consisting of an intrinsically incompressible solid skeleton and an intrinsically incompressible fluid phase. We formulate the poroelastic problem using a stabilized three‐field finite‐strain framework, following the general approach of Berger et al. [11]. The resulting formulation treats the solid displacement u, nominal fluid flux W, and pore pressure p as independent primary variables.

#### 2.1.1 Kinematics

Let $\Omega_{0}\subset {\mathbb{R}}^{3}$ denote the reference configuration of the myocardium, and let Ω(t) denote its current configuration at time t. The deformation map is defined as

(1) x=χ(X,t)=X+u(X,t),

where $X\in \Omega_{0}$ and x∈Ω(t) are the material positions in the reference and current configurations, respectively. The deformation gradient, right Cauchy–Green deformation tensor, and Jacobian are, respectively,

(2) $$F=\frac{\partial \chi }{\partial X}=I+\text{Grad}\;u,\;C=F^{T}F,\;J=\det F>0.$$

Here, Grad(⋅) and Div(⋅) denote the gradient and divergence operators with respect to the reference coordinate X, whereas ∇(⋅) and div(⋅) denote the corresponding spatial operators. The Green–Lagrange strain tensor is defined as

(3) $$E=\frac{1}{2}\left(C-I\right).$$

#### 2.1.2 Conservation of Mass

Let ϕ denote the fluid volume fraction in the current configuration. The solid and fluid volume fractions are therefore 1−ϕ and ϕ, respectively. The reference configuration is attached to the solid skeleton and the local mass balance for the solid phase is given as

(4) $$\frac{\partial }{\partial t}\left[J\left(1-\phi \right)\rho^{s}\right]=0,$$

where $\rho^{s}$ is the intrinsic density of the solid constituent. Let $v^{s}$ and $v^{f}$ denote the spatial velocities of the solid and fluid phases, respectively. The spatial fluid volume flux relative to the solid skeleton is defined as

(5) $$z=\phi \left(v^{f}-v^{s}\right).$$

and the nominal fluid flux per unit reference area is obtained through the Piola transformation

(6) $$W=JF^{-1}z.$$

Correspondingly, the local fluid‐phase mass balance in the reference configuration is given by

(7) $$\frac{\partial }{\partial t}\left(J{\phi \rho }^{f}\right)+\mathrm{Div}\left(\rho^{f}W\right)=J\rho^{f}s,$$

where $\rho^{f}$ is the intrinsic fluid density and s is the volumetric fluid source per unit current volume. Assuming both constituents are incompressible, the intrinsic densities $\rho^{s}$ and $\rho^{f}$ are constant. Dividing Equations (4) and (7) by their respective intrinsic densities and summing the resulting equations gives the mixture mass balance

(8) $$\frac{\partial }{\partial t}\left[J\left(1-\phi \right)+\mathrm{J\phi}\right]+\mathrm{Div}\;W=\mathrm{Js},$$

which further reduces to:

(9) $$\dot{J}+\mathrm{Div}\;W=\mathrm{Js}.$$

Based on the exact finite‐deformation identity for the rate of the Jacobian, that is, $\dot{J}=\mathrm{Div}\left(JF^{-1}\dot{u}\right)$, the mixture mass balance equation can be expressed as

(10) $$\mathrm{Div}\left(JF^{-1}\dot{u}+W\right)=\mathrm{Js}.$$

Equation (10) follows directly from the phase‐wise mass balances, constituent incompressibility, mixture saturation, and the Piola transformation. No infinitesimal‐strain or constitutive linearization is introduced in its derivation.

#### 2.1.3 Darcy Flow

With fluid body forces neglected, the spatial Darcy relation is given by

(11) $$k^{-1}z+\nabla p=0,$$

where k is the spatial hydraulic permeability tensor that is related to the referential hydraulic permeability tensor $\kappa_{0}$ by

(12) $$k=J^{-1}{\mathrm{F\kappa}}_{0}F^{T}.$$

Using $z=J^{-1}\mathrm{FW}$ and $\nabla p=F^{-T}\text{Grad}\;p$, Equation (11) can be pulled back to the reference configuration and is given by

(13) $$\kappa_{0}^{-1}W+\text{Grad}\;p=0.$$

#### 2.1.4 Balance of Linear Momentum

Under the quasi‐static assumption and in the absence of body forces, the balance of linear momentum in the reference configuration is given by

(14) $$\mathrm{Div}\;P_{\mathrm{tot}}=0.$$

In Equation (14), the total stress is given by $P_{\mathrm{tot}}=P_{\mathrm{eff}}-\mathrm{Jp}F^{-T}$ based on the assumption of an isotropic pore‐pressure contribution and the effective first Piola–Kirchhoff stress carried by the myocardial solid skeleton is given by

(15) $$P_{\mathrm{eff}}=P_{p}+P_{a}.$$

where $P_{p}$ and $P_{a}$ are the passive and active components, respectively. The momentum balance can therefore be written as

(16) $$\mathrm{Div}\left(P_{p}+P_{a}-\mathrm{Jp}F^{-T}\right)=0.$$

#### 2.1.5 Passive Myocardial Constitutive Model

The passive stress component is obtained from the strain‐energy density $\Psi_{p}$ as

(17) $$P_{p}=\frac{\partial \Psi_{p}}{\partial F}.$$

Here, we use the Guccione transversely isotropic hyperelastic constitutive model [12] that has the following strain‐energy density function:

(18) $$\Psi_{p}=\frac{C_{G}}{2}\left[\exp\left(Q\right)-1\right],$$

where 

(19)

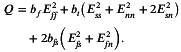




In the above, $C_{G}$ is a stress‐like scaling parameter, while $b_{f}$, $b_{t}$, and $b_{\mathrm{fs}}$ are dimensionless parameters controlling the stiffness in the fiber, transverse, and fiber–transverse shear directions, respectively. Based on a local orthonormal material coordinate system is defined by the myocardial fiber, sheet, and sheet‐normal directions, components of the Green–Lagrange strain tensor in Equation (19) are given as

(20) $$E_{\mathrm{ij}}=e_{i}\cdot {\mathrm{Ee}}_{j},\;i,j\in \left\{f,s,n\right\}.$$

The passive material parameters used in the simulations are summarized in Table 1.

**TABLE 1:** Parameters of the Guccione passive myocardial constitutive model.

Parameter	Description	Value	Unit
$C_{G}$	Passive stress scaling parameter	130	kPa
$b_{f}$	Fiber stiffness parameter	29	—
$b_{t}$	Transverse stiffness parameter	26.6	—
$b_{\mathrm{fs}}$	Fiber–transverse shear parameter	13.3	—

#### 2.1.6 Active Myocardial Constitutive Model

The active stress component is assumed to act along the myocardial fiber direction and is described using a modified time‐varying elastance active‐stress model [13].

(21) $$P_{a}=F\left(S_{\mathrm{act}}f_{0}⊗f_{0}\right),$$

where the scalar active fiber stress is calculated as follows:

(22) $$S_{\mathrm{act}}=T_{\max}C_{t}\left(\hat{t},X\right)\frac{{\mathrm{Ca}}_{0}^{2}}{{\mathrm{Ca}}_{0}^{2}+{\mathrm{ECa}}_{50}^{2}\left(\lambda_{f}\right)}.$$

In Equation (22), $T_{\max}$ is the maximum isometric active tension, ${\mathrm{Ca}}_{0}$ is the prescribed peak intracellular calcium concentration, and $C_{t}$ is the temporal activation function. The length‐dependent calcium sensitivity is given by

(23) $${\mathrm{ECa}}_{50}\left(\lambda_{f}\right)=\frac{{\left({\mathrm{Ca}}_{0}\right)}_{\max}}{\sqrt{\exp\left[B\left(l_{s}-l_{0}\right)\right]-1}},$$

where ${\left({\mathrm{Ca}}_{0}\right)}_{\max}$ is the maximum peak intracellular calcium concentration, B controls the length dependence of calcium sensitivity and $l_{0}$ is the sarcomere length below which no active tension develops. The current sarcomere length is calculated as $l_{s}=\lambda_{f}l_{r}$ where $l_{r}$ is the stress‐free sarcomere length and the myocardial fiber stretch is defined $\lambda_{f}=\sqrt{f_{0}\cdot {\mathrm{Cf}}_{0}}$. To avoid a numerical singularity when $l_{s}$ approaches $l_{0}$, the implementation uses

(24) $$l_{s}-l_{0}\leftarrow \max\left(l_{s}-l_{0},0.002\;\mathrm{\mu m}\right)$$

when evaluating Equation (23). The temporal activation function with $\hat{t}$ denoting time elapsed since activation is given by

(25) $$C_{t}\left(\hat{t}\right)=\left\{\begin{array}{ll} 0, & \hat{t}\leq 0,\\ \frac{1}{2}\left[1-\cos\left(\frac{\pi \hat{t}}{t_{0}}\right)\right], & 0<\hat{t}<t_{\text{trans}},\\ \frac{1}{2}\left[1-\cos\left(\frac{\pi t_{\text{trans}}}{t_{0}}\right)\right]\exp\left[-\frac{\hat{t}-t_{\text{trans}}}{\tau }\right], & \hat{t}\geq t_{\text{trans}}, \end{array}\right.$$

where $t_{0}$ controls the contraction time scale, $t_{\text{trans}}$ denotes the transition from contraction to relaxation, and τ is the exponential relaxation time constant. The active material parameters are summarized in Table 2.

**TABLE 2:** Parameters of the modified time‐varying elastance active‐stress model.

Parameter	Description	Value	Unit
$T_{\max}$	Maximum isometric active tension	135.7	kPa
${\mathrm{Ca}}_{0}$	Peak intracellular calcium concentration	4.35	μM
${\left({\mathrm{Ca}}_{0}\right)}_{\max}$	Maximum peak intracellular calcium concentration	4.35	μM
B	Length‐dependence parameter	4.75	${\mathrm{\mu m}}^{-1}$
$l_{0}$	Sarcomere length at zero active tension	1.60	μm
$l_{r}$	Stress‐free sarcomere length	1.85	μm
$t_{0}$	Contraction time parameter	275	ms
$t_{\text{trans}}$	Contraction‐to‐relaxation transition time	320	ms
τ	Exponential relaxation time constant	30	ms

#### 2.1.7 Three‐Field Boundary‐Value Problem

The resulting finite‐deformation poroelastic problem is expressed in terms of the three independent fields (u,W,p) with the following governing equations given in the reference configuration

(26a) $$\begin{array}{l} \mathrm{Div}\;P_{\mathrm{tot}}=0,\;\text{in}\;\Omega_{0}, \end{array}$$

(26b) $$\begin{array}{l} \kappa_{0}^{-1}W+\text{Grad}\;p=0,\;\text{in}\;\Omega_{0}, \end{array}$$

(26c) $$\begin{array}{l} \dot{J}+\mathrm{Div}\;W=\mathrm{Js},\;\text{in}\;\Omega_{0}. \end{array}$$

These equations are supplemented by the mechanical boundary conditions.

(27a) $$\begin{array}{l} u=u_{D},\;\mathrm{on}\;\Gamma_{u}^{0}, \end{array}$$

(27b) $$\begin{array}{l} P_{\mathrm{tot}}N=T_{N},\;\mathrm{on}\;\Gamma_{t}^{0}, \end{array}$$

For the fluid problem considered here, no pore‐pressure Dirichlet condition is prescribed on the external myocardial boundary (i.e., Γp0=0). The entire external boundary is instead treated as impermeable to the nominal Darcy flux with the homogeneous normal‐flux condition

(28) $$W\cdot N=0\;\mathrm{on}\;\partial \Omega_{0}=\Gamma_{W}^{0}$$

where N is the outward unit normal to the reference myocardial boundary. This condition constrains only the normal component of the nominal Darcy flux and therefore does not suppress tangential intramyocardial transport. The coronary arterial and venous pressures are introduced through the distributed volumetric source and sink terms described in Section 2.2, rather than through pore‐pressure boundary conditions.

### 2.2 Computational Modeling Framework

Figure 1 shows a schematic of the computational modeling framework. The poroelastic formulation is used to describe the mechanical behavior and intramyocardial blood transport of the LV and is coupled to a closed‐loop lumped‐parameter circulation model. The 0D circulation comprises resistive and compliant vascular compartments, unidirectional cardiac valves, and systemic vascular pathways, consistent with the general structure of established closed‐loop cardiovascular models [14, 15, 16, 17]. The compartmental pressures, volumes, and flow rates are governed by a system of ordinary differential equations, as detailed in Appendix A. Through this coupling strategy, the LV cavity pressure predicted by the lumped‐parameter model is applied to the endocardial surface ($\Gamma_{t}^{0}$) as the pressure‐traction boundary condition defined in Equation (27b). The displacement normal to the truncated basal plane ($\Gamma_{u}^{0}$) is constrained by imposing $u_{z}=0$, while the in‐plane basal displacement remains unconstrained. Together with this basal condition, five global Lagrange‐multiplier constraints are used to remove the remaining rigid translations and rotations. No additional external traction is prescribed on the epicardium, which therefore satisfies the natural zero‐traction condition. For the pore‐fluid problem, the entire external surface of the LV ($\partial \Omega_{0}$) is treated as impermeable by imposing W⋅N=0. This condition is enforced weakly through a penalty term acting only on the normal component of the nominal Darcy flux. No pore‐pressure Dirichlet condition is prescribed on the external myocardial boundary; the coronary arterial and venous pressures instead enter through the distributed volumetric source and sink terms described below.

**FIGURE 1:**
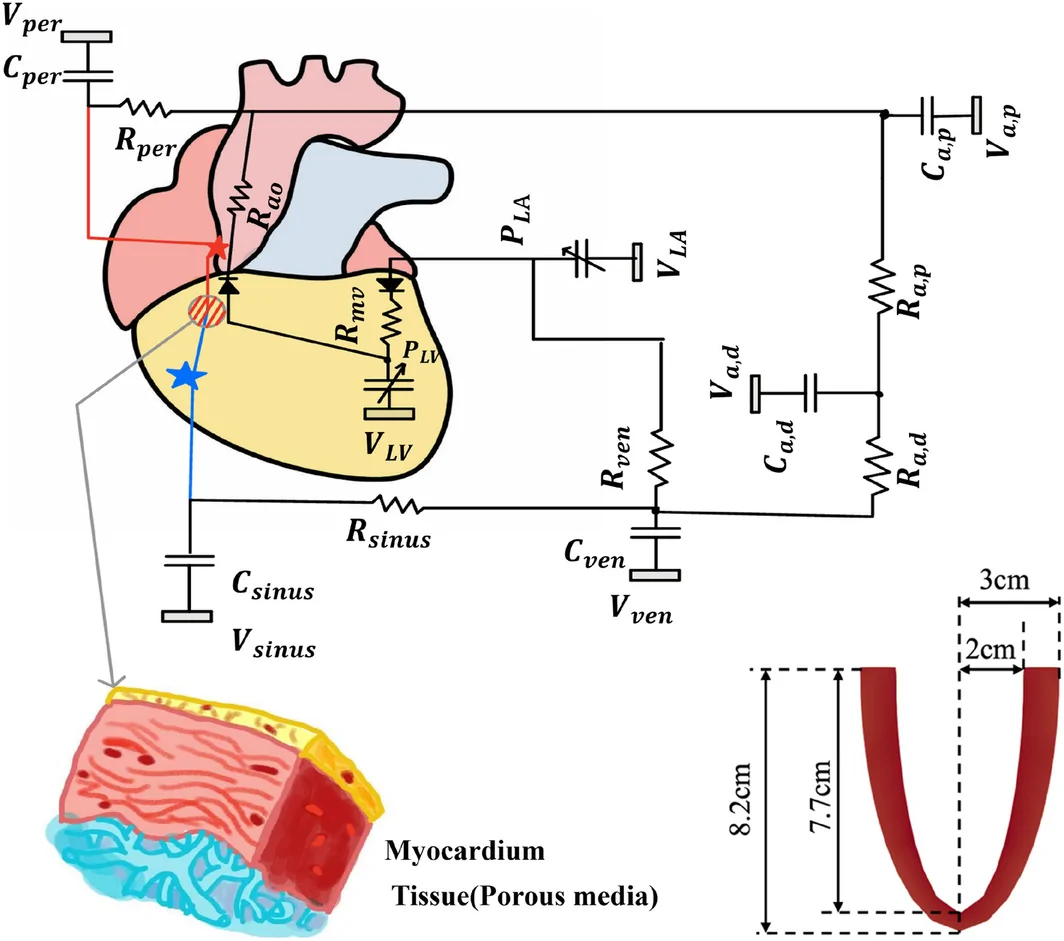
Schematic of the computational framework consisting of the poroelastic left ventricle coupled with a closed‐loop lumped‐parameter circulation model.

In addition, the computational framework accounts for myocardial perfusion, with blood supplied from the upstream coronary arterial compartment and drained through the coronary sinus compartment. This coupling is accomplished by defining the myocardial fluid source per unit current volume in Equation (26c) as follows:

(29) $$s=\beta_{a}^{\mathrm{eff}}\left(p_{a}-p\right)-\beta_{v}\left(p-p_{v}\right),$$

where p is the myocardial pore pressure, while $p_{a}$ and $p_{v}$ denote the pressures in the upstream coronary arterial and coronary sinus compartments, respectively. The parameters $\beta_{a}^{\mathrm{eff}}$ and $\beta_{v}$ represent the effective arterial‐to‐myocardial and myocardial‐to‐venous exchange conductances, respectively. To account for the reduction in coronary microvascular conductance caused by myocardial compression during systole [18], the effective arterial exchange conductance is defined as a function of LV cavity pressure $p_{\mathrm{LV}}$ by

(30) $$\beta_{a}^{\mathrm{eff}}=\beta_{a}g_{\text{comp}}\left(p_{\mathrm{LV}}\right),$$

where $\beta_{a}$ is the baseline arterial exchange conductance and $g_{\text{comp}}$ is a dimensionless compression factor given as

(31) $$g_{\text{comp}}\left(p_{\mathrm{LV}}\right)=g_{\min}+\frac{1-g_{\min}}{1+\exp\left[\left(p_{\mathrm{LV}}-p_{50}\right)/k_{p}\right]}.$$

Here, $g_{\min}$ is the minimum fraction of the baseline arterial conductance during strong systolic compression, $p_{50}$ is the midpoint pressure of the transition, and $k_{p}$ controls the transition width. The parameters are prescribed to be $g_{\min}=0.42$, $p_{50}=35\;\text{mmHg}$, and $k_{p}=5\;\text{mmHg}$. The value of $g_{\min}$ is selected based on the approximate ratio between reported human left‐coronary diastolic and systolic microvascular resistances [18]. At low LV pressure during diastole, $g_{\text{comp}}\to 1$ and $\beta_{a}^{\mathrm{eff}}\to \beta_{a}$, thereby approximately preserving the baseline diastolic conductance. At high LV pressure during systole, $g_{\text{comp}}\to g_{\min}$, representing the reduction in arterial conductance caused by myocardial compression.

The coronary arterial inflow and venous outflow communicated to the lumped‐parameter circulation are calculated by integrating the corresponding distributed source and sink terms over the reference myocardium:

(32) $$Q_{\mathrm{cor},\text{in}}=\int_{\Omega_{0}}J\beta_{a}g_{\text{comp}}\left(p_{\mathrm{LV}}\right)\left(p_{a}-p\right)\;d\Omega_{0},$$

(33) $$Q_{\mathrm{cor},\text{out}}=\int_{\Omega_{0}}J\beta_{v}\left(p-p_{v}\right)\;d\Omega_{0}.$$

The same arterial and venous exchange expressions are used in both the poroelastic governing equations and the lumped‐parameter circulation. As such, the lumped‐parameter circulatory model and the LV poroelastic model are coupled bidirectionally and consistently.

### 2.3 Numerical Method

The coupled poroelastic problem was solved using a stabilized three‐field finite element formulation. Let $T_{h}$ be a conforming, shape‐regular tetrahedral partition of the reference myocardial domain $\Omega_{0}$. The solid displacement u, nominal Darcy flux W, and pore pressure p finite element spaces are defined as

(34) $$\begin{array}{l} V_{h}=\left\{{\left.v_{h}\in {\left[H^{1}\left(\Omega_{0}\right)\right]}^{3}:v_{h}\right|}_{K}\in {\left[P_{1}\left(K\right)\right]}^{3},\;\forall K\in T_{h}\right\},\\ Z_{h}=\left\{{\left.w_{h}\in {\left[H^{1}\left(\Omega_{0}\right)\right]}^{3}:w_{h}\right|}_{K}\in {\left[P_{1}\left(K\right)\right]}^{3},\;\forall K\in T_{h}\right\},\\ Q_{h}=\left\{{\left.q_{h}\in L^{2}\left(\Omega_{0}\right):q_{h}\right|}_{K}\in P_{0}\left(K\right),\;\forall K\in T_{h}\right\}. \end{array}$$

Accordingly, continuous piecewise‐linear approximations are employed for both displacement and nominal Darcy flux, whereas the pore pressure is represented by a discontinuous piecewise‐constant approximation (i.e., $P_{1}-P_{1}-P_{0}$ discretization). Denoting $\Delta t=t^{n}-t^{n-1}$ as time‐step, the deformation‐dependent mass balance is discretized in time using the backward Euler method:

(35) $$\frac{J_{h}^{n}-J_{h}^{n-1}}{\Delta t}+\mathrm{Div}\;W_{h}^{n}=J_{h}^{n}s_{h}^{n},$$

where $s_{h}^{n}$ is the myocardial perfusion source term evaluated at the current time step. The source term includes the pressure‐dependent arterial conductance described in Section 2.2.

At each time step, the physical poroelastic unknowns are $\left(u_{h}^{n},W_{h}^{n},p_{h}^{n}\right)$. Five spatially constant Lagrange multipliers are appended to the discrete system to remove the rigid‐body modes described in Section 2.2. The complete discrete state is therefore

(36) $$y_{h}^{n}=\left(u_{h}^{n},W_{h}^{n},p_{h}^{n},\lambda_{h}^{n}\right)\in V_{h}\times Z_{h}\times Q_{h}\times {\mathbb{R}}^{5},$$

where $\lambda_{h}^{n}\in {\mathbb{R}}^{5}$ contains the auxiliary rigid‐body Lagrange multipliers. These multipliers do not constitute an additional poroelastic field.

For all test functions $\left(v_{h},w_{h},q_{h}\right)\in V_{h}\times Z_{h}\times Q_{h}$, the three poroelastic fields satisfy

(37a) $$\int_{\Omega_{0}}\left(P_{\mathrm{eff},h}^{n}-J_{h}^{n}p_{h}^{n}F_{h}^{-T,n}\right):\nabla_{0}v_{h}\;\mathrm{dX}-{ℒ}_{u}^{n}\left(v_{h}\right)+C_{\mathrm{rb}}\left(v_{h},\lambda_{h}^{n}\right)=0,$$

(37b) $$\int_{\Omega_{0}}\kappa_{0}^{-1}W_{h}^{n}\cdot w_{h}\;\mathrm{dX}-\int_{\Omega_{0}}p_{h}^{n}\;\mathrm{Div}\;w_{h}\;\mathrm{dX}+{ℬ}_{W}\left(W_{h}^{n},p_{h}^{n};w_{h}\right)=0,$$

(37c) $$\begin{array}{cc} & \int_{\Omega_{0}}q_{h}\frac{J_{h}^{n}-J_{h}^{n-1}}{\Delta t}\;\mathrm{dX}+\int_{\Omega_{0}}q_{h}\;\mathrm{Div}\;W_{h}^{n}\;\mathrm{dX}+S_{h}\left(\frac{p_{h}^{n}-p_{h}^{n-1}}{\Delta t},q_{h}\right)\\ & \;-\int_{\Omega_{0}}J_{h}^{n}s_{h}^{n}q_{h}\;\mathrm{dX}=0. \end{array}$$

Here, $P_{\mathrm{eff},h}^{n}$ is the effective first Piola–Kirchhoff stress associated with the passive and active myocardial material models. The functional ${ℒ}_{u}^{n}$ represents the LV cavity‐pressure loading, while $C_{\mathrm{rb}}$ denotes the contribution of the five rigid‐body Lagrange multipliers. Together with the strongly imposed basal condition $u_{z}=0$, these multiplier equations eliminate the six rigid‐body modes without fixing an arbitrary point in the myocardium.

Because no pore‐pressure Dirichlet condition is prescribed, Equation (37b) contains no boundary term involving a prescribed pressure $p_{D}$. Integration by parts of the pressure‐gradient term gives the consistent boundary contribution

(38) $$\int_{\Gamma_{W}^{0}}p_{h}^{n}w_{h}\cdot N\;{\mathrm{dA}}_{0}.$$

The homogeneous normal‐flux condition in Equation (28) is imposed weakly by adding a penalty acting only on the normal component of the nominal Darcy flux. The complete Darcy boundary contribution is 

(39)

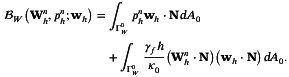




Here, h is the local cell diameter, $\kappa_{0}$ is the reference hydraulic permeability, and $\gamma_{f}=10^{3}$ is the dimensionless normal‐flux penalty parameter. Because the penalty acts only on $W_{h}\cdot N$, tangential intramyocardial transport remains unconstrained.

The continuous piecewise‐linear approximation of the displacement and nominal Darcy‐flux fields, together with a discontinuous piecewise‐constant approximation of the pore pressure, does not in general satisfy the discrete inf–sup condition without stabilization [11, 19]. Consequently, the discrete problem may exhibit nonphysical element‐to‐element oscillations in the pressure field, particularly in low‐permeability regimes [11, 20]. To control these spurious pressure modes, the semi‐discrete mass‐balance equation is augmented by a consistent interior‐facet pressure‐jump stabilization term following the approach of Berger et al. [11]. Denoting ${ℱ}_{h}^{i}$ the set of interior facets. The pressure jump for an interior facet $F=K^{+}\cap K^{-}$ is defined as

(40) $$\left[\;\left[p_{h}\right]\;\right]=p_{h}^{+}-p_{h}^{-}$$

and the stabilization operator in Equation 37 (c) is given as follows:

(41) $$S_{h}\left(r_{h},q_{h}\right)=\gamma \sum_{F\in {ℱ}_{h}^{i}}h_{F}\int_{F}\left[\;\left[r_{h}\right]\;\right]\left[\;\left[q_{h}\right]\;\right]\;\mathrm{dS},$$

where $h_{F}$ is a characteristic facet size and $\gamma =\frac{\gamma_{0}}{P_{\mathrm{ref}}}=10^{-5}$ is the stabilization coefficient, and $P_{\mathrm{ref}}$ is a reference pressure introduced to ensure dimensional consistency. Applying Equation (41) to the backward difference in pressure gives

(42) $$S_{h}\left(\frac{p_{h}^{n}-p_{h}^{n-1}}{\Delta t},q_{h}\right)=\gamma \sum_{F\in {ℱ}_{h}^{i}}h_{F}\int_{F}\left[\;\left[\frac{p_{h}^{n}-p_{h}^{n-1}}{\Delta t}\right]\;\right]\left[\;\left[q_{h}\right]\;\right]\;\mathrm{dS}.$$

This term suppresses nonphysical element‐to‐element pressure oscillations without introducing an element‐wise pressure‐gradient term. In particular, because $p_{h}$ and $q_{h}$ belong to $P_{0}\left(K\right)$, their gradients vanish within each element; therefore, neither $\nabla_{0}p_{h}$ nor $\nabla_{0}q_{h}$ appears in the implemented formulation.

At each time step, the closed‐loop circulation model was first advanced to obtain the target LV cavity volume. An outer scalar iteration then adjusted the LV cavity pressure until the cavity volume computed from the finite‐element deformation matched the target volume. For each trial LV pressure, the displacement, nominal Darcy‐flux, and pore‐pressure fields were solved monolithically using Newton's method. Spatial discretization was performed using ${\mathrm{CG}}_{1}$–${\mathrm{CG}}_{1}$–${\mathrm{DG}}_{0}$ finite element spaces on a tetrahedral mesh comprising 1118 elements. The discretized system contained 3439 degrees of freedom, including five real‐valued Lagrange multipliers used to constrain rigid‐body motion. Time discretization was performed using the backward Euler scheme with a time‐step size of 1ms. The resulting nonlinear algebraic system was solved using Newton's method, with the consistent Jacobian computed by automatic differentiation in FEniCS. Newton iterations were terminated when either the absolute or relative residual norm fell below $10^{-8}$ or $10^{-9}$, respectively, with a maximum of 200 iterations per time step. All simulations were implemented in FEniCS 2019.1.0, and the linearized systems were solved using the MUMPS direct solver.

### 2.4 Simulation Cases

#### 2.4.1 Validation Case

The computational framework was first validated against experimental observations of perfusion‐induced stiffening where we performed simulations on an isolated and non‐contractile LV under varying cavity and perfusion pressures. Specifically, the base was fixed, and no‐flux BCs were applied on the endocardial and epicardial surfaces. Pressure–volume (P–V) relations were obtained with perfusion pressure varying between 0 and 60 mmHg and LV cavity pressure varying between 0 and 20 mmHg, which replicates the experimental conditions in McCulloch et al. [21].

#### 2.4.2 Baseline Case

After validating the passive case, we simulated a *baseline case*, where the LV model was coupled to the circulatory model as described in Section 2.2. Similarly, no‐flux boundary conditions were prescribed on both the endocardial and epicardial surfaces for the pore fluid. The base was also constrained in the apex‐base direction, and the remaining rigid‐body modes were removed using the five global Lagrange‐multiplier constraints described in Section 2.2. LV cavity pressure is applied to endocardium surface as a traction boundary condition. No additional external traction was prescribed on the epicardium. The periodic steady state is defined as relative $L_{2}$ cycle‐to‐cycle errors for LV volume($\epsilon_{V}$), LV pressure($\epsilon_{P}$), and coronary flow($\epsilon_{Q}$) and require $\max\left(\epsilon_{V},\epsilon_{P},\epsilon_{Q}\right)<10^{-3}$. Results shown are those that reach time periodic steady state.

#### 2.4.3 Coronary Sinus Occlusion Cases

Next, we performed simulations to investigate the impact of CSO on coronary perfusion. To simulate different degrees of CSO, we increase the coronary sinus resistance ($R_{\text{sinus}}$) to 1×, 100×, 1000×, and 10,000× of its value in the *baseline case* to reflect a decrease in the coronary sinus diameter. Model parameters are tabulated in Appendix A.

## 3 Results

### 3.1 Passive Case Validation

Figure 2 shows the model‐predicted LV pressure–volume relationship under a perfusion pressure of 0 and 60 mmHg. As perfusion pressure is increased, the change in LV cavity volume at a given LV cavity pressure is decreased, indicating a reduction in compliance (or an increase in stiffness) of the myocardium. Quantitative comparison shows close agreement between the model predictions and experimental measurements [21] in both perfusion conditions. The root mean square error between predicted and measured LV pressure is approximately 0.95 mmHg for the non‐perfused case (0 mmHg) and 0.75 mmHg for the 60 mmHg perfusion condition.

**FIGURE 2:**
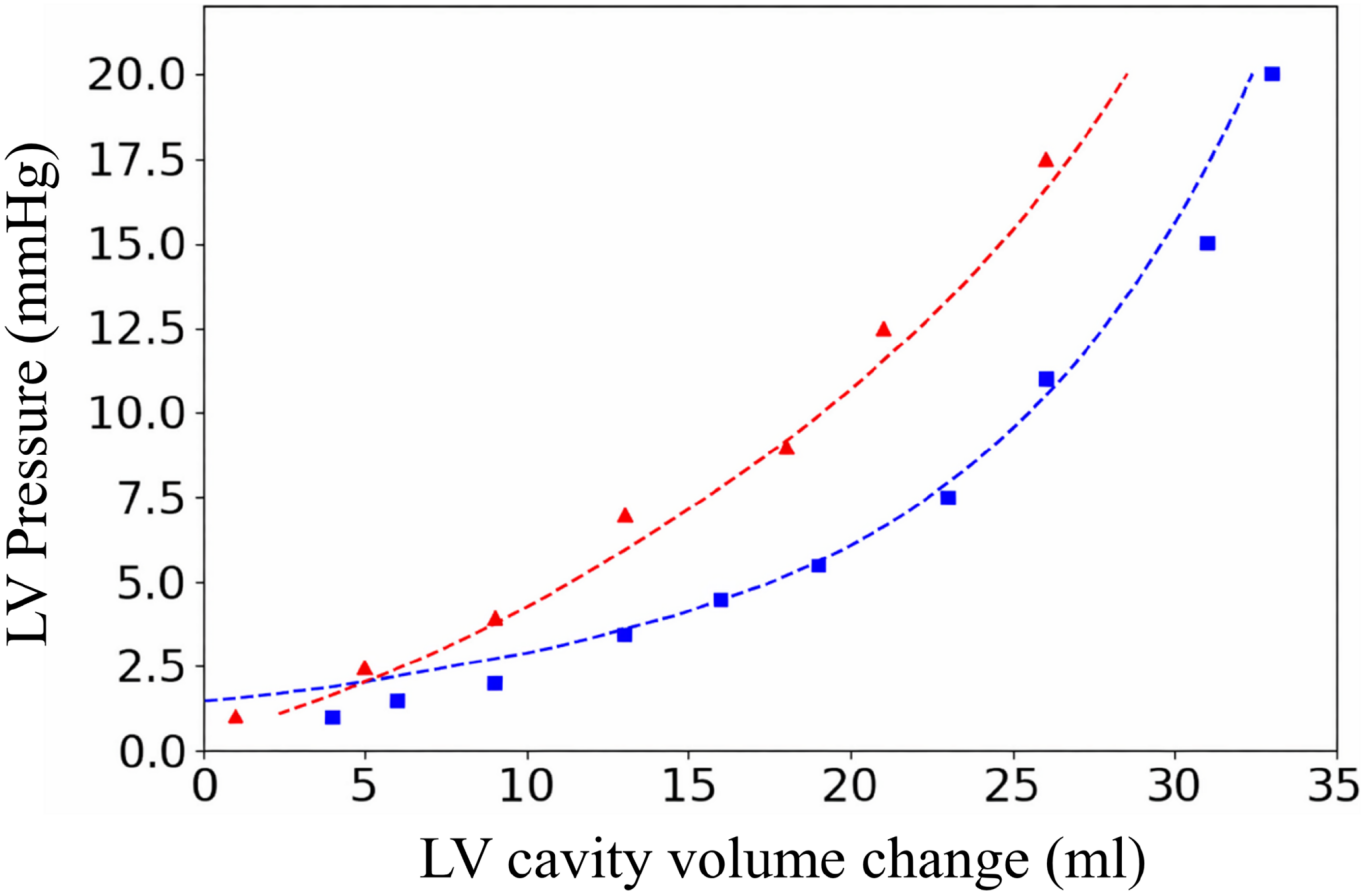
Comparison of LV pressure‐volume relationship between model predictions (dashed lines) and experimental data (symbols) [21] at perfusion pressures of 0 mmHg (blue) and 60 mmHg (red).

### 3.2 Baseline Simulation Case

Figure 3 shows the model‐predicted waveforms of the coronary flow rate, LV cavity pressure, wall volume as well as the pressure‐volume loop over a cardiac cycle at a steady time‐periodic state in the *baseline* simulation. Coronary flow rate is reduced during early systole before recovering and reaching its maximum during diastole (Figure 3a), which compares well with experiments [22] (Figure 3b).

**FIGURE 3:**
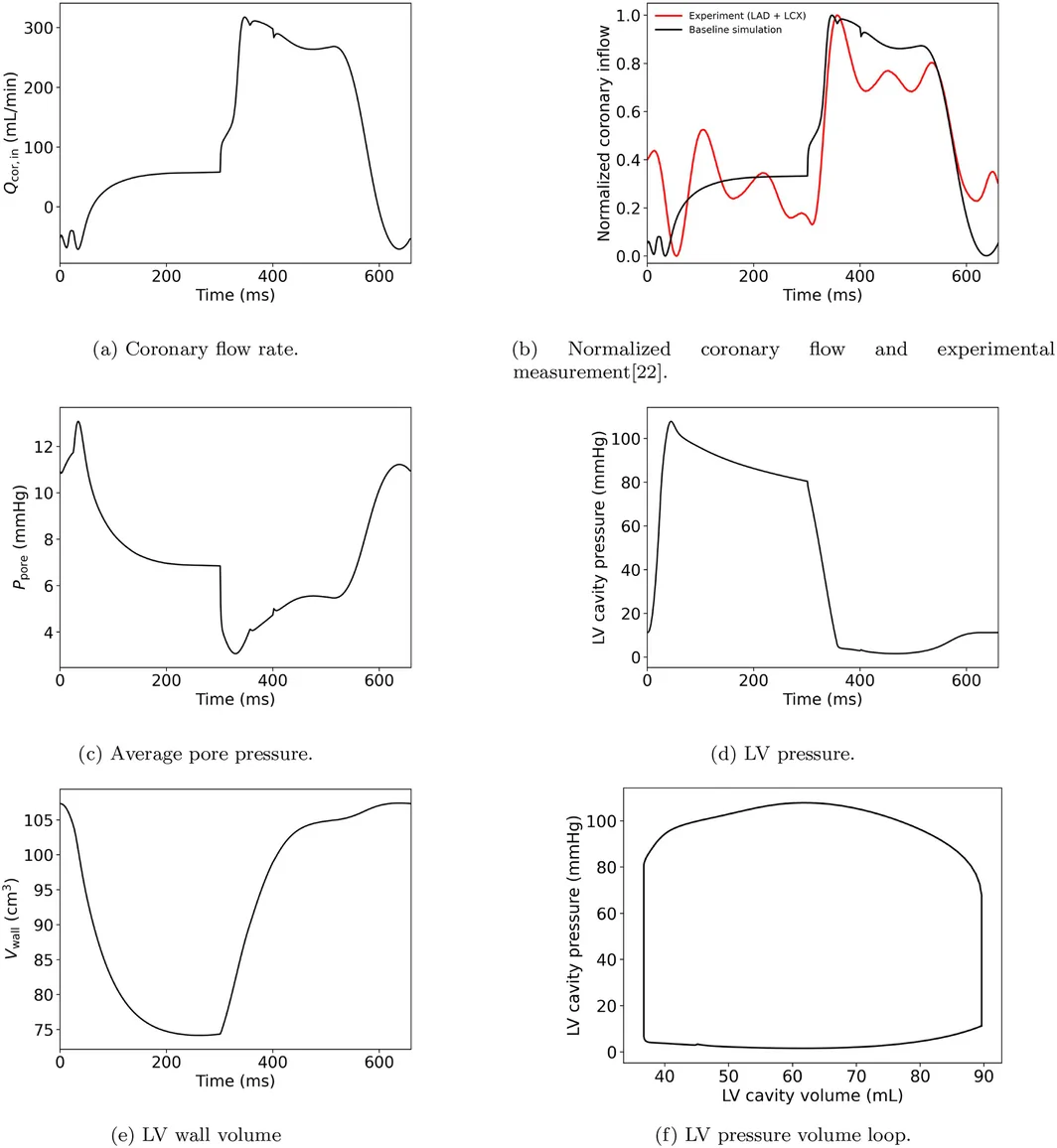
(a) Coronary flow rate dynamics (b) comparison between normalized model prediction coronary flow and experiment measurement [22] (c) Average pore pressure (d) LV pressure (e) LV wall volume (f) LV pressure‐volume loop over a cardiac cycle at steady time‐periodic state in the *baseline* simulation.

The coronary flow rate waveform also mirrored the phases found in the pore pressure and LV pressure waveforms. (Figure 3c,d) Specifically, the lowest coronary flow rate corresponds to the rapid upstroke in LV pressure that compresses the wall during early systole. The rapid reduction in coronary flow rate is also temporally aligned with the rapid increase in pore pressure during the period between the end of diastole and the beginning of systole (Figure 3c). The recovered coronary flow during early diastole also corresponds to the recovery of LV wall volume during diastole (Figure 3e). The pressure volume loop shows an ejection fraction of 59.91%, end diastolic volume of 89.107 mL and end systolic volume of 35.720 mL (Figure 3f).

Figure 4 shows the transmural variation of myocardial perfusion during a cardiac cycle in the *baseline* simulation. Fluid flux across the mid‐wall (positive towards the epicardium) rises to its peak value at elevated LV cavity pressure during systole, then changes sign at early diastole and reaches its minimum value before the beginning of ventricular filling. A change in the sign of flux indicates a reversal of the direction of fluid motion across the LV mid‐wall. The temporal variation of fluid flux is consistent with the evolving transmural gradient of the pore pressure (Figure 4a). Specifically, a larger increase in pore pressure in the endocardial region during systole establishes an outward gradient that drives the fluid toward the epicardium. Subsequently, with the LV pressure starting to decrease rapidly, the pore pressure gradient is reversed, and the fluid flux becomes inward. The relative transmural fluid volume change further shows a more pronounced reduction in fluid volume fraction in the subendocardial region during systole, compared to the subepicardial region (Figure 4b). Subendocardial perfusion also exhibits a stronger diastolic predominance, characterized by greater systolic flow suppression and more pronounced early‐systolic reverse flow than that in the subepicardial region (Figure 4c).

**FIGURE 4:**
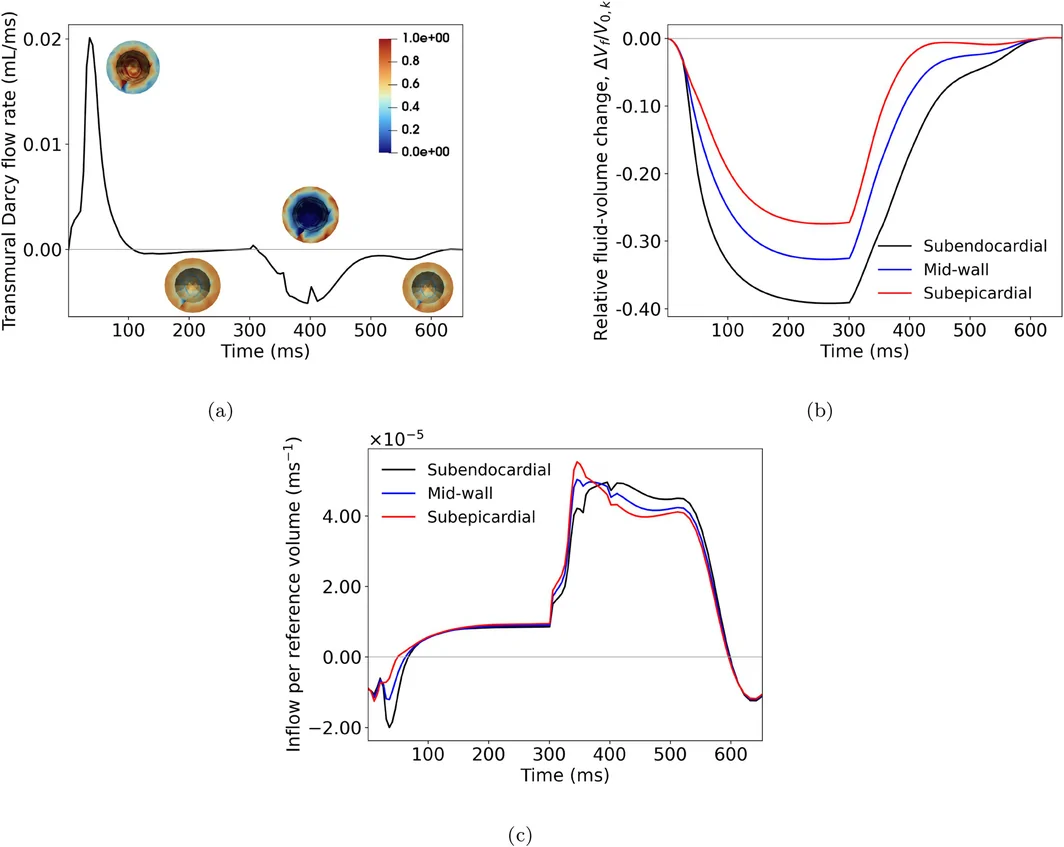
Model prediction of transmural variation of (a) Darcy flux across LV mid layer. Inset shows transmural variation of normalized pore pressure, (b) relative fluid volume change, (c) normalized regional perfusion flow in LV wall over a cardiac cycle at steady time‐periodic state.

### 3.3 Coronary Sinus Occlusion Simulations

Figure 5 shows the effects of increasing the coronary‐sinus resistance $R_{\text{sinus}}$, which reflects different degrees of CSO. An increase in the resistance leads to an increase in the pressure of the coronary sinus (Figure 5a) as well as a reduction in the mean coronary flow rate and its pulsatility (Figure 5b). Compared to the *baseline* case, mean coronary flow is reduced by 13.84% (from 114.27 to 98.45mL/min) and its pulsatility is reduced by 31.4% (from 388.83 to 266.61mL/min) when $R_{\text{sinus}}$ is increased from 100 to $10^{6}\;Pa\cdot \mathrm{ms}/\mathrm{mL}$. Pore pressure is also increased with coronary‐sinus resistance (Figure 5c). LV end diastolic volume and systolic cavity pressure are slightly reduced when coronary sinus resistance is increased to $10^{6}\;Pa\cdot \mathrm{ms}/\mathrm{mL}$ (Figure 5d) and the LV wall volume is also increased with CSO (Figure 5e).

**FIGURE 5:**
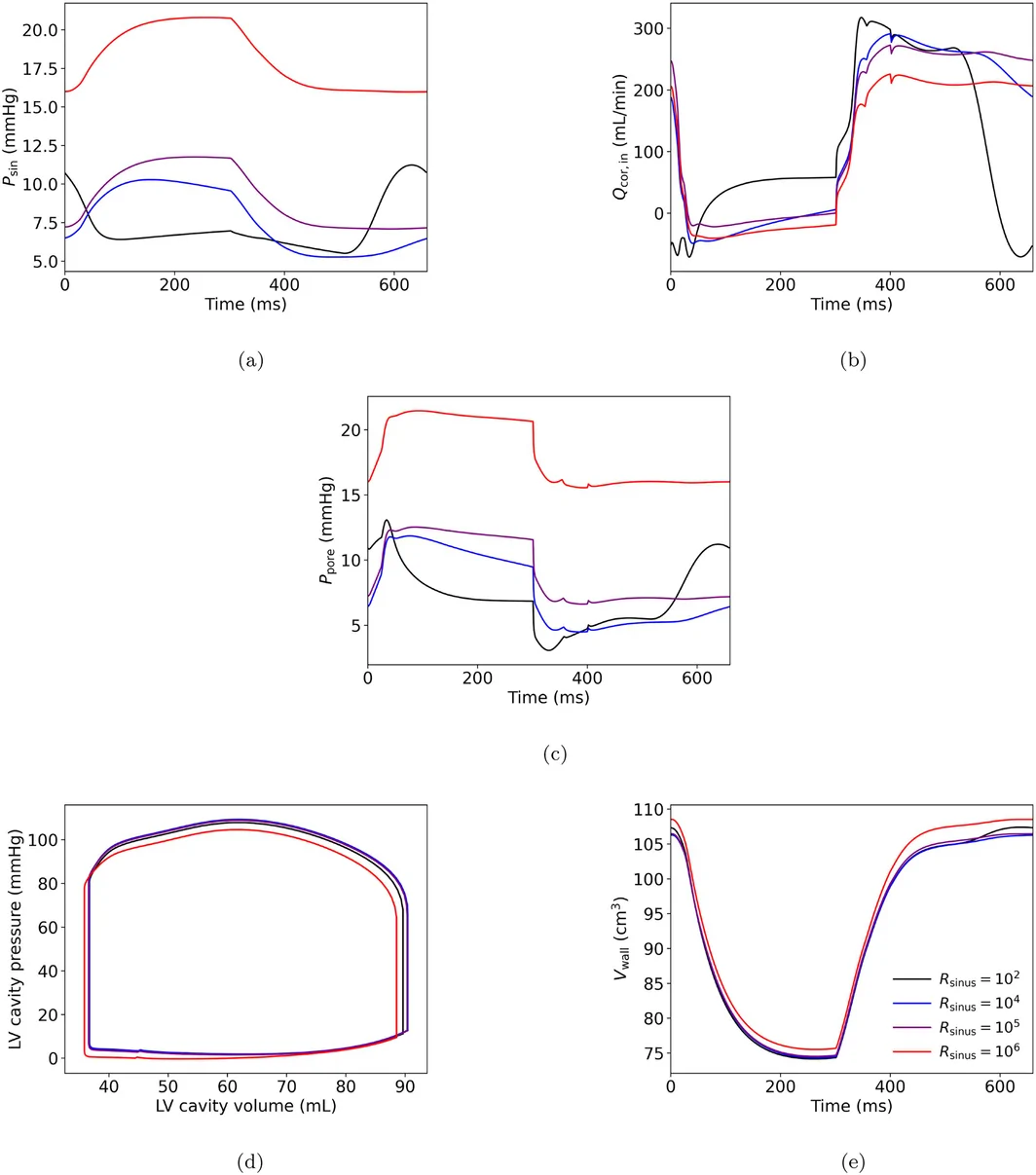
Effects of increasing coronary sinus resistance on the waveform of (a) coronary sinus pressure; (b) coronary flow rate; (c) average pore pressure; (d) LV pressure‐volume loop and (e) LV wall volume over a cardiac cycle.

## 4 Discussion

We have developed a computational modeling framework in which the LV is described as a poroelastic media to simulate regional myocardial perfusion and the effects of CSO. The computational framework explicitly accounts for tissue deformation–induced pore pressure evolution and fluid transport across the myocardial wall, allowing myocardial wall perfusion dynamics and myocardial wall mechanics to interact bidirectionally. In this framework, myocardial perfusion is also coupled bidirectionally with a lumped‐parameter 0D representation of both the systemic and coronary circulations. While spatially resolved 1D and 3D vascular models can capture pulse‐wave propagation, wave reflection, and distributed vascular properties [23, 24], the closed‐loop 0D formulation, which has been used in other studies [14, 15, 16, 17, 25], provides an appropriate balance between physiological representation and computational efficiency.

### 4.1 Comparison of Baseline Case With Experiments

We show that the poroelastic LV model can reproduce isolated heart experiments showing perfusion‐induced stiffening of the myocardium, where an increase in myocardial wall perfusion reduces the compliance of the LV [21]. As shown in Figure 3b simulations from the computational modeling framework can reproduce key physiological features found in the measurements of coronary flow rate waveform, including a reduction in coronary flow rate at early‐systole associated with the rise in LV pressure, and a peak in the coronary flow rate at diastole [22]. Transmural differences in myocardial perfusion are consistent with experimental and physiological observations showing stronger systolic compression of the subendocardium and a diastolic perfusion gradient favoring the subendocardial layer [26, 27, 28]. The model also predicts an LV wall volume waveform that is comparable with that found in the experiments. Specifically, the smallest myocardial wall volume (normalized with its largest value) that occurs during systole (∼0.71) is comparable to the measured value (∼0.8) found in a canine experiment [29].

### 4.2 Effects of Coronary Sinus Occlusion

CSO, which was conceived several decades ago as a treatment for ischemia [30], is emerging recently as treatment for patients with refractory angina [31]. By elevating the coronary sinus pressure, this treatment is hypothesized to help redistribute flow to the ischemic region, which is supported by a recent computer modeling study [2]. However, this treatment may also cause venous congestion, with potential downstream consequences such as venous engorgement and myocardial edema [32]. These features are found in the simulation results of CSO, which show that mean coronary flow rate is decreased, intramyocardial pore pressure is increased, and the myocardium swells (0.93%–2.8%) with increasing degree of occlusion. By occluding the coronary sinus, pressure in that vessel is elevated, which promotes pore‐pressure accumulation within the tissue and reduces the pressure gradient between coronary arteries and the myocardium that decreases the mean coronary flow rate. The degree of swelling in the myocardium is also comparable to that measured in a canine model of acute coronary sinus hypertension [33], which reported an increase in the tissue water content by 2.84%.

### 4.3 Comparison With Other Models of Myocardial Perfusion

Poroelastic tissue models have been developed to simulate myocardial perfusion by treating the myocardium as a fluid‐saturated porous solid, where Darcy‐type fluid transport is coupled to tissue deformation and stress. In this class of models, perfusion is controlled by deformation‐dependent pore‐pressure dynamics, which enables the direct quantification of fluid accumulation (edema) and its mechanical consequences. Representative developments range from early poroelastic descriptions aimed at explaining perfusion‐induced stiffening in isolated hearts to finite deformation and thermodynamically consistent formulations that establish a rigorous constitutive basis for large‐strain poromechanics and its numerical implementation, and more recent multiscale models that integrate porous‐tissue perfusion with coronary vessel representations and circulation components [7, 8, 9, 10].

Despite these advances, existing perfusion–mechanics models often adopt either (i) prescribed coronary pressures/flows that limit the ability to test vascular interventions, or (ii) coupling strategies that reduce feedback from perfusion to mechanics to improve computational efficiency. To address these issues, the framework here introduces a unified, bidirectionally coupled poroelastic–lumped parameter model that connects finite‐deformation LV wall mechanics to a closed‐loop systemic circulation and an explicit coronary inflow–outflow pathway, including coronary sinus drainage. This structure allows hemodynamic boundary conditions to be generated endogenously by the circulation model and updated in response to ventricular deformation, while the resulting coronary inflow/outflow is simultaneously determined by tissue‐level pore‐pressure evolution. Consequently, the model can reproduce key physiological features (e.g., diastolic‐dominant coronary inflow and early‐systolic flow suppression) while also predicting deformation‐driven pore‐pressure transients and wall‐volume changes consistent with experimental observations. This coupling enables mechanistic interrogation of venous‐side effects on myocardial perfusion as discussed above.

### 4.4 Limitations in Scope and Fidelity

There are several limitations associated with the model. *First*, the current formulation adopts a single‐compartment poroelastic description of the myocardium and does not explicitly distinguish among arterial, capillary, and venous vascular compartments. This homogenized representation may limit the model's ability to resolve compartment‐specific perfusion dynamics. *Second*, the coronary circulation is represented using a lumped‐parameter model rather than a one‐dimensional vascular network or a three‐dimensional fluid‐dynamics formulation, which may restrict the capture of fine‐scale physiological features such as spatial heterogeneity and wave propagation. A coronary network and a regional perfusion source term will be integrated to our future multiscale computational framework. *Third*, the pore‐fluid phase represents homogenized blood transport through the intramyocardial microvasculature rather than a separate interstitial‐fluid compartment. Consequently, the present model does not explicitly account for transvascular fluid exchange, colloid osmotic effects, or lymphatic drainage. These processes will need to be incorporated to investigate myocardial interstitial edema in greater physiological detail.

### 4.5 Conclusions

In this study, we developed a poroelastic–lumped parameter computational framework that integrates myocardial tissue mechanics with coronary and systemic hemodynamics. The model can reproduce key physiological features of perfusion‐induced stiffening, as well as dynamic changes in coronary flow rate and myocardial wall observed in experimental studies. Application of the framework to simulate CSO revealed that elevated coronary sinus resistance led to an increase in pore pressure that lead to fluid retention and myocardial swelling.

## Funding

This work was supported by the National Institutes of Health (HL160997) and National Science Foundation (2222066).

## Ethics Statement

The authors have nothing to report.

## Conflicts of Interest

The authors declare no conflicts of interest.

## Data Availability

The data supporting the findings of this study are available from the corresponding author upon reasonable request. The code is available at https://github.com/anhaowei/poroelastic‐computational‐framework.

## Appendix A Systemic Circulation Model

The poroelastic LV model is coupled to a closed‐loop 0D lumped‐parameter systemic circulation. The circulation comprises resistive–compliant vascular compartments and unidirectional valves, with volumes updated from flow balance at each time step (Figure 1). Compartmental volume conservation is expressed as follows:

(A1a)
$$\frac{{\mathrm{dV}}_{\mathrm{LA}}}{\mathrm{dt}}=q_{\mathrm{ven}}-q_{\mathrm{mv}},\;\frac{{\mathrm{dV}}_{\mathrm{LV}}}{\mathrm{dt}}=q_{\mathrm{mv}}-q_{\mathrm{ao}},$$

(A1b)
$$\frac{{\mathrm{dV}}_{\mathrm{ap}}}{\mathrm{dt}}=q_{\mathrm{ao}}-q_{\mathrm{ap}}-q_{\mathrm{per}},\;\frac{{\mathrm{dV}}_{\mathrm{per}}}{\mathrm{dt}}=q_{\mathrm{per}}-q_{\mathrm{cor},\text{in}},$$

(A1c)
$$\frac{{\mathrm{dV}}_{\mathrm{ven}}}{\mathrm{dt}}=q_{\mathrm{ad}}-q_{\mathrm{ven}}+q_{\sin},\;\frac{{\mathrm{dV}}_{\sin}}{\mathrm{dt}}=q_{\mathrm{cor},\text{out}}-q_{\sin},$$

(A1d)
$$\frac{{\mathrm{dV}}_{\mathrm{ad}}}{\mathrm{dt}}=q_{\mathrm{ap}}-q_{\mathrm{ad}}.$$

Here $V_{\mathrm{LA}},V_{\mathrm{LV}},V_{\mathrm{ap}},V_{\mathrm{ad}},V_{\mathrm{per}},V_{\sin},V_{\mathrm{ven}}$ denote the volumes of left atrium, LV, proximal/distal systemic arteries, coronary branches, CS, and systemic veins. Coronary inflow ($q_{\mathrm{cor},\text{in}}$) and outflow ($q_{\mathrm{cor},\text{out}}$) are directly provided by the poroelastic LV model.

Unidirectional valve flows were pressure‐driven:

(A2)
$$q_{\mathrm{ao}}=\max\left(0,\frac{P_{\mathrm{LV}}-P_{\mathrm{ap}}}{R_{\mathrm{ao}}}\right),\;q_{\mathrm{mv}}=\max\left(0,\frac{P_{\mathrm{LA}}-P_{\mathrm{LV}}}{R_{\mathrm{mv}}}\right).$$

Vascular segments follow the Ohm's law:

(A3)
$$q_{\mathrm{ap}}=\frac{P_{\mathrm{ap}}-P_{\mathrm{ad}}}{R_{\mathrm{ap}}},\;q_{\mathrm{ad}}=\frac{P_{\mathrm{ad}}-P_{\mathrm{ven}}}{R_{\mathrm{ad}}},\;q_{\mathrm{ven}}=\frac{P_{\mathrm{ven}}-P_{\mathrm{LA}}}{R_{\mathrm{ven}}},$$

(A4)
$$q_{\mathrm{per}}=\frac{P_{\mathrm{ap}}-P_{\mathrm{per}}}{R_{\mathrm{per}}},\;q_{\sin}=\frac{P_{\sin}-P_{\mathrm{ven}}}{R_{\sin}}.$$

Compartmental pressures are computed from compliance relations:

(A5)
$$P_{\mathrm{ap}}=\frac{V_{\mathrm{ap}}-V_{\mathrm{ap},0}}{C_{\mathrm{ap}}},\;P_{\mathrm{ad}}=\frac{V_{\mathrm{ad}}-V_{\mathrm{ad},0}}{C_{\mathrm{ad}}},\;P_{\mathrm{ven}}=\frac{V_{\mathrm{ven}}-V_{\mathrm{ven},0}}{C_{\mathrm{ven}}},$$

(A6)
$$P_{\mathrm{per}}=\frac{V_{\mathrm{per}}-V_{\mathrm{per},0}}{C_{\mathrm{per}}},\;P_{\sin}=\frac{V_{\sin}-V_{\sin,0}}{C_{\sin}}.$$

Unstressed volumes ($V_{i,0}$) and compliances ($C_{i}$) characterizes the passive properties of each compartment, while resistances ($R_{i}$) represents hydraulic resistance of valves, arteries, veins, and coronary pathways (Table A1).

**TABLE A1:** Model parameters and initial values.

Parameter	Units	Value
$R_{\mathrm{ao}}$	mmHg⋅ms/mL	15.0
$R_{\mathrm{ven}}$	mmHg⋅ms/mL	0.75
$R_{\mathrm{mv}}$	mmHg⋅ms/mL	15.0
$R_{a,p}$	mmHg⋅ms/mL	135.0
$R_{a,d}$	mmHg⋅ms/mL	785.0
$R_{\mathrm{per}}$	mmHg⋅ms/mL	35,000
$R_{\text{sinus}}$	mmHg⋅ms/mL	0.74
$C_{a,p}$	mL/mmHg	1.35
$C_{\mathrm{ven}}$	mL/mmHg	16.2
$C_{a,d}$	mL/mmHg	1.76
$C_{\mathrm{per}}$	mL/mmHg	4.05
$C_{\text{sinus}}$	mL/mmHg	24.3
$V_{\mathrm{LA}}$	mL	12
$V_{\mathrm{LV}}$	mL	112
$V_{a,p}$	mL	740
$V_{a,d}$	mL	100
$V_{\mathrm{per}}$	mL	35
$V_{\mathrm{ven}}$	mL	3580
$V_{\text{sinus}}$	mL	35
$\kappa_{0}$	cm/(mmHg⋅s)	9.3
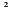
$\beta_{a}$	mL/(mmHg⋅ms)	$9.3\times 10^{-6}$
$\beta_{v}$	mL/(mmHg⋅ms)	$1.1\times 10^{-3}$

## References

[1] J. A. E. Spaan , “Mechanical Determinants of Myocardial Perfusion,” Basic Research in Cardiology 90, no. 2 (1995): 89–102. 10.1007/BF00789439 7646422

[2] H. Wang , L. Fan , J. S. Choy , G. S. Kassab , and L. C. Lee , “Mechanisms of Coronary Sinus Reducer for Treatment of Myocardial Ischemia: In Silico Study,” Journal of Applied Physiology 136, no. 5 (2024): 1157–1169. 10.1152/japplphysiol.00910.2023 PMC11368528 38511210

[3] T. J. Ford , E. Redwood , and E. Chuah , “Coronary Sinus Reduction: Can Device‐Based Therapy Improve Coronary Microvascular Function?,” Circulation: Cardiovascular Interventions 17, no. 1 (2024): e013831. 10.1161/CIRCINTERVENTIONS.123.013831 38227698

[4] M. A. Biot , “General Theory of Three‐Dimensional Consolidation,” Journal of Applied Physics 12, no. 2 (1941): 155–164.

[5] B. R. Simon , “Multiphase Poroelastic Finite Element Models for Soft Tissue Structures,” Applied Mechanics Reviews 45, no. 6 (1992): 191–218.

[6] J. M. Huyghe , D. H. van Campen , T. Arts , and R. M. Heethaar , “A Two‐Phase Finite Element Model of the Diastolic Left Ventricle,” Journal of Biomechanics 24, no. 7 (1991): 527–538. 10.1016/0021-9290(91)90286-v 1880137

[7] A. N. Cookson , J. Lee , C. Michler , et al., “A Novel Porous Mechanical Framework for Modelling the Interaction Between Coronary Perfusion and Myocardial Mechanics,” Journal of Biomechanics 45, no. 5 (2012): 850–855. 10.1016/j.jbiomech.2011.11.026 PMC3334269 22154392

[8] D. Chapelle , J.‐F. Gerbeau , J. Sainte‐Marie , and I. E. Vignon‐Clementel , “A Poroelastic Model Valid in Large Strains With Applications to Perfusion in Cardiac Modeling,” Computational Mechanics 46, no. 1 (2010): 91–101.

[9] N. A. Barnafi Wittwer , S. Di Gregorio , L. Dedè , P. Zunino , C. Vergara , and A. Quarteroni , “A Multiscale Poromechanics Model Integrating Myocardial Perfusion and the Epicardial Coronary Vessels,” SIAM Journal on Applied Mathematics 82, no. 4 (2022): 1167–1193.

[10] S. H. Richardson , J. Mackenzie , N. Thekkethil , et al., “Cardiac Perfusion Coupled With a Structured Coronary Network Tree,” Computer Methods in Applied Mechanics and Engineering 428 (2024): 117083.

[11] L. Berger , R. Bordas , D. Kay , and S. Tavener , “A Stabilized Finite Element Method for Finite‐Strain Three‐Field Poroelasticity,” Computational Mechanics 60, no. 1 (2017): 51–68. 10.1007/s00466-017-1381-8 PMC6979590 32025072

[12] J. M. Guccione , A. D. McCulloch , and L. K. Waldman , “Passive Material Properties of Intact Ventricular Myocardium Determined From a Cylindrical Model,” Journal of Biomechanical Engineering 113, no. 1 (1991): 42–55. 10.1115/1.2894084 2020175

[13] S. M. Shavik , S. Wall , J. Sundnes , et al., “Computational Modeling Studies of the Roles of Left Ventricular Geometry, Afterload, and Muscle Contractility on Myocardial Strains in Heart Failure With Preserved Ejection Fraction,” Journal of Cardiovascular Translational Research 14, no. 6 (2021): 1131–1145. 10.1007/s12265-021-10130-y PMC9305326 33928526

[14] M. Hirschvogel , M. Bassilious , L. Jagschies , S. M. Wildhirt , and M. W. Gee , “A Monolithic 3D–0D Coupled Closed‐Loop Model of the Heart and the Vascular System: Experiment‐Based Parameter Estimation for Patient‐Specific Cardiac Mechanics,” International Journal for Numerical Methods in Biomedical Engineering 33, no. 8 (2017): e2842. 10.1002/cnm.2842 27743468

[15] C. M. Augustin , M. A. F. Gsell , E. Karabelas , et al., “A Computationally Efficient Physiologically Comprehensive 3D–0D Closed‐Loop Model of the Heart and Circulation,” Computer Methods in Applied Mechanics and Engineering 386 (2021): 114092. 10.1016/j.cma.2021.114092 PMC7611781 34630765

[16] F. Regazzoni , M. Salvador , P. C. Africa , M. Fedele , L. Dedè , and A. Quarteroni , “A Cardiac Electromechanical Model Coupled With a Lumped‐Parameter Model for Closed‐Loop Blood Circulation,” Journal of Computational Physics 457 (2022): 111083.

[17] R. Piersanti , F. Regazzoni , M. Salvador , et al., “3D–0D Closed‐Loop Model for the Simulation of Cardiac Biventricular Electromechanics,” Computer Methods in Applied Mechanics and Engineering 391 (2022): 114607.

[18] H. Seligman , S. S. Nijjer , T. P. van de Hoef , et al., “Phasic Flow Patterns of Right Versus Left Coronary Arteries in Patients Undergoing Clinical Physiological Assessment,” EuroIntervention 17, no. 15 (2022): 1260–1270. 10.4244/EIJ-D-21-00189 PMC9724998 34338643

[19] L. Berger , R. Bordas , D. Kay , and S. Tavener , “Stabilized Lowest‐Order Finite Element Approximation for Linear Three‐Field Poroelasticity,” SIAM Journal on Scientific Computing 37, no. 5 (2015): A2222–A2245.

[20] J. B. Haga , H. Osnes , and H. P. Langtangen , “On the Causes of Pressure Oscillations in Low‐Permeable and Low‐Compressible Porous Media,” International Journal for Numerical and Analytical Methods in Geomechanics 36, no. 12 (2012): 1507–1522.

[21] A. D. McCulloch , P. J. Hunter , and B. H. Smaill , “Mechanical Effects of Coronary Perfusion in the Passive Canine Left Ventricle,” American Journal of Physiology. Heart and Circulatory Physiology 262, no. 2 (1992): H523–H530. 10.1152/ajpheart.1992.262.2.H523 1539711

[22] J. S. Choy , L. Fan , Y. Awakeem , et al., “Acute Effects of Mechanical Dyssynchrony on Left Ventricular Function and Coronary Perfusion,” Frontiers in Bioengineering and Biotechnology 13 (2025): 1630854. 10.3389/fbioe.2025.1630854 PMC12463955 41017940

[23] J. P. Mynard and J. J. Smolich , “One‐Dimensional Haemodynamic Modeling and Wave Dynamics in the Entire Adult Circulation,” Annals of Biomedical Engineering 43, no. 6 (2015): 1443–1460. 10.1007/s10439-015-1313-8 25832485

[24] F. Caforio , C. M. Augustin , J. Alastruey , M. A. F. Gsell , and G. Plank , “A Coupling Strategy for a First 3D–1D Model of the Cardiovascular System to Study the Effects of Pulse Wave Propagation on Cardiac Function,” Computational Mechanics 70, no. 4 (2022): 703–722. 10.1007/s00466-022-02206-6 PMC9477941 36124206

[25] N. Westerhof , J.‐W. Lankhaar , and B. E. Westerhof , “The Arterial Windkessel,” Medical & Biological Engineering & Computing 47, no. 2 (2009): 131–141. 10.1007/s11517-008-0359-2 18543011

[26] D. J. Duncker , A. Koller , D. Merkus , and J. M. Canty, Jr. , “Regulation of Coronary Blood Flow in Health and Ischemic Heart Disease,” Progress in Cardiovascular Diseases 57, no. 5 (2015): 409–422. 10.1016/j.pcad.2014.12.002 PMC5856234 25475073

[27] I. A. Vermeltfoort , P. G. Raijmakers , M. Lubberink , et al., “Feasibility of Subendocardial and Subepicardial Myocardial Perfusion Measurements in Healthy Normals With ^15^O‐Labeled Water and Positron Emission Tomography,” Journal of Nuclear Cardiology 18, no. 4 (2011): 650–656. 10.1007/s12350-011-9375-y PMC3143328 21519976

[28] M. Kondo , K. Kawata , A. Azuma , et al., “Relationship Between Coronary Blood Flow Velocity Waveform and Transmural Distribution of Myocardial Blood Flow in Coronary Artery,” Japanese Heart Journal 40, no. 6 (1999): 783–790. 10.1536/jhj.40.783 10737562

[29] H. Ashikaga , B. A. Coppola , K. G. Yamazaki , F. J. Villarreal , J. H. Omens , and J. W. Covell , “Changes in Regional Myocardial Volume During the Cardiac Cycle: Implications for Transmural Blood Flow and Cardiac Structure,” American Journal of Physiology ‐ Heart and Circulatory Physiology 295, no. 2 (2008): H610–H618. 10.1152/ajpheart.00107.2008 PMC2519196 18515651

[30] G. S. Kassab , J. A. Navia , K. March , and J. S. Choy , “Coronary Venous Retroperfusion: An Old Concept, a New Approach,” Journal of Applied Physiology 104, no. 5 (2008): 1266–1272. 10.1152/japplphysiol.00063.2008 18292303

[31] M. J. Foley , C. A. Rajkumar , F. Ahmed‐Jushuf , et al., “Coronary Sinus Reducer for the Treatment of Refractory Angina (Orbita‐Cosmic): A Randomised, Placebo‐Controlled Trial,” Lancet 403, no. 10436 (2024): 1543–1553. 10.1016/S0140-6736(24)00256-3 38604209

[32] A. Ido , S. Kawashima , T. Yamashita , and M. Yokoyama , “Coronary Sinus Occlusion Enhances Coronary Collateral Flow and Reduces Subendocardial Ischemia,” American Journal of Physiology ‐ Heart and Circulatory Physiology 280, no. 3 (2001): H1361–H1368. 10.1152/ajpheart.2001.280.3.H1361 11179085

[33] J. W. Pratt , E. R. Schertel , S. L. Schaefer , et al., “Acute Transient Coronary Sinus Hypertension Impairs Left Ventricular Function and Induces Myocardial Edema,” American Journal of Physiology. Heart and Circulatory Physiology 271, no. 3 (1996): H834–H841. 10.1152/ajpheart.1996.271.3.H834 8853315

